# Evaluating audio-visual falls prevention messages with community-dwelling older people using a World Café forum approach

**DOI:** 10.1186/s12877-019-1344-3

**Published:** 2019-12-09

**Authors:** Lex D. de Jong, Jacqueline Francis-Coad, Chris Wortham, Terry P. Haines, Dawn A. Skelton, Tammy Weselman, Anne-Marie Hill

**Affiliations:** 1 School of Physiotherapy and Exercise Science, Faculty of Health Sciences, Curtin University, Bentley, WA Australia; 2 The University of Notre Dame Australia, School of of Physiotherapy, Fremantle, Australia; 3 The University of Notre Dame Australia, School of Arts and Sciences, Fremantle, Australia; 4 School of Primary and Allied Health Care, Monash University, Frankston, VIC Australia; 5 School of Health and Life Sciences, Glasgow Caledonian University, Glasgow, Scotland, UK

**Keywords:** Accidental falls, Community-based participatory research, Consumer health information, Health behavior, Qualitative research

## Abstract

**Background:**

Falls risk increases sharply with older age but many older people are unaware or underestimate their risk of falling. Increased population-based efforts to influence older people’s falls prevention behavior are urgently needed. The aim of this study was to obtain a group of older people’s collective perspectives on newly developed prototypes of audio-visual (AV) falls prevention messages, and evaluate changes in their falls prevention behaviour after watching and discussing these.

**Methods:**

A mixed-method study using a community World Café forum approach.

**Results:**

Although the forum participants (*n* = 38) mostly responded positively to the three AV messages and showed a significant increase in their falls prevention capability and motivation after the forum, the participants collectively felt the AV messages needed a more inspirational call to action. The forum suggested this could be achieved by means of targeting the message and increasing the personal connection. Participants further suggested several alternatives to online falls prevention information, such as printed information in places in the community, as a means to increase opportunity to seek out falls prevention information.

**Conclusions:**

Falls prevention promotion messages need to be carefully tailored if they are to be more motivating to older people to take action to do something about their falls risk. A wider variety of revised and tailored AV messages, as one component of a community-wide falls prevention campaign, could be considered in an effort to persuade older people to take decisive action to do something about their falls risk.

**Trial registration:**

This study was registered prospectively: NCT03154788. Registered 11 May 2017.

## Background

Fall rates and injurious falls among older people are escalating worldwide [1–3]. Currently, one in three adults over the age of 65 fall each year [4], with many of these falls resulting in serious injury [5] or even death [6]. Because falls are such a ubiquitous problem, efforts to successfully translate falls prevention evidence into practice are urgently needed [7].

Undertaking fall prevention activities that target identified falls risk factors is recommended in several guidelines e.g. [7, 8]) but to be effective this requires older people to adopt and enact them in their daily lives. A recent systematic review [9] has shown that interventions such as exercise for strength and balance reduces falls risk. However, many older people show no interest in participating in these falls prevention activities [10]. For example, despite having concerns about falling and a desire to do something about it, a majority of community-dwelling older people surveyed reported they were not likely to participate in programmes that were offered to manage concerns about falls [11]. Also, the proportion of people aged over 65 years in a representative sample undertaking strength training, balance training and tai chi was only 12.1, 6.2 and 2.8%, respectively [12]. These findings show that, despite the availability of multiple falls prevention resources and programs to increase older people’s engagement in falls prevention activities(e.g. [13]), uptake of falls prevention activities among older people is low. This is in part explained by the fact that many older people, even those who have had a fall [14], feel that falls prevention activities are not suitable for them [15] but rather “*better for others than for me*” [16] because they do not perceive themselves to be at risk of falling [17, 18]. Indeed, the label of being at risk of falling is often rejected by older people because it is negatively associated with aging and loss of independence [19]. Other large studies have found that older people lack knowledge about falls and falls prevention [20–22] which is also not conducive towards engagement in falls prevention activities.

Concepts of health behaviour change explain that when an individual lacks awareness and knowledge about a health condition and its potential consequences they are unlikely to take preventive action to reduce the risk to their health [23]. Additionally, if individuals do not see potential benefit to engaging in healthy behaviours such as exercise, they are unlikely to undertake these type of health behaviours. There is, however, limited research that has evaluated how older people initially become aware about falls risk increasing with age and what encourages them to seek out falls prevention resources.

In order to raise knowledge and awareness about the benefits of undertaking falls prevention strategies, community-dwelling older people who participated in a previously held World Café forum suggested that targeting all ages to work together to prevent falls and raising broad community awareness and knowledge about falls such as in shopping centres and libraries was important [24]. Results of another qualitative study suggested that promoting falls prevention in beauty salons could be useful [25]. Older people also suggested that they needed to be portrayed in the media as ‘active healthy people’ [24]. These findings prompted local researchers to collaborate with a reference panel of older people in an effort to create audio-visual (AV) messages that promoted falls prevention in the community in the context of a positive view of aging. The design of these AV messages was also based on recommendations from the literature such as showing real people doing something [26], providing tips on how to successfully implement the recommended actions and convincing people that they are personally at risk [27]. It was envisaged that these AV messages could potentially be used as one component of a future community-wide falls prevention campaign that could operate through television, social media and health systems. As recommended in the literature [28, 29] community-dwelling older people’s perspectives regarding these prototype AV messages were subsequently explored using focus groups. The findings of this study [30] suggested that for some older people the messages had increased their falls prevention capability (i.e. falls risk awareness and falls prevention knowledge) and motivation to take up falls prevention strategies, but not for several others. Potential changes in the participants’ overall falls prevention capability and motivation were not assessed.

## Methods

### Aim and design

To obtain a broad community perspective on three prototype AV falls prevention messages we undertook a mixed-method design [31] using a community World Café forum approach. This forum approach enables larger groups, compared with most traditional conversational methods such as focus groups, to participate together. Evolving rounds of dialogue with varying combinations of participants were enabled while at the same time they remained part of the larger group as a means to explore new insights into questions that deeply matter to participants [32, 33]. During this event the participants’ collective perspective of the messages was sought and pre-post changes in the participants’ falls prevention capability (knowledge and awareness) and motivation (intentions) towards undertaking falls prevention activities were evaluated.

### Participants and setting

A purposeful sampling method was utilised with the aim to recruit independently living community-dwelling older adults over the age of 50. Participants were further eligible for participation if they had sufficient English language skills (self-assessed) to join in the table conversations and were able to provide written informed consent. The forum was announced in various local media (e.g. a radio show, newsletters and websites of older peoples’ organizations).

### Questionnaire development, pretesting and reliability

Prior to the study, and according to principles of questionnaire design [34] and past falls prevention work [35], a bespoke questionnaire was constructed to suit the context of the forum. All questions were formulated at the seventh-grade English literacy level [36, 37]. The part of the questionnaire that assessed the extent of the participant’s agreement or disagreement with statements about their capability and motivation towards undertaking falls prevention activities (collected pre-forum using nine Likert-scale [38] items) was based on the constructs of the Behaviour Change Wheel and Theoretical Domains Framework approach, a theoretical psychological model of behaviour change [23]. Another part assessed participants’ reaction and response to the content, appeal and quality of each individual AV message (collected post-forum using 18 Likert-scale items). The main part of the questionnaire contained 11 multiple-choice falls prevention knowledge statements, each with one key and two distractors. These statements reflected the falls facts that were presented in the different AV messages. It also contained a vertical visual analogue scale (VAS) asking participants about their level of intent to engage in healthy ageing activities that are also good for falls prevention.

After construction, a pretest of the procedural feasibility and data quality of the questionnaire was conducted. Six community-dwelling older people were asked to complete the questionnaire and identify any issues during a “talk through” [34] session. The questionnaire was revised based on their feedback. Subsequently, a panel of six content experts and another seven community-dwelling older people were asked to rate the overall quality of the questionnaire. Most raters strongly agreed or agreed that the questions were relevant to the study (*n* = 12, 92%), easy to understand (*n* = 13, 100%) and had adequate font-size (*n* = 12, 92%), layout (*n* = 11, 85%) and length (*n* = 9, 75%). To calculate the overall index of agreement for all five statements combined, the five Likert-scale categories were reduced to three categories (*strongly agree* and *agree*, *undecided, disagree* and *strongly disagree*). The overall mean of all kappa values for all 13 coder pairs [39] was 0.65.

Because the 11 multiple-choice falls prevention knowledge statements (to assess capability) and VAS scores (to asssess motivation) were planned to be used for the pre-post evaluation during the forum, the test-retest reliability of these items was established prior to the study. It was determined a-priori that items with a test-retest reliability below 0.5 would not be used. Forty-six study participants recruited during another study [30] completed these parts and once more (with the questions in the reverse order) after a 20-min morning tea. Agreement across the multiple-choice items between test and retest occasions ranged between 0.29 and 0.84. Six of the items had kappa values between 0.57 and 0.84. Five items had kappa values ranging between 0.29 and 0.39, suggesting that these items were not appropriate to assess the participants’ capability. As such, these items were not included in the composite ‘knowledge score’. The intraclass correlation coefficient (ICC, two-way mixed, consistency) for the participants’ mean VAS scores was 0.95 (95% CI 0.90 to 0.97).

### World Café forum areas of conversation and procedure

The forum took place in a venue in a central location of a large city. The forum was conducted using the World Café’s seven integrated principles: (i) setting context; (ii) creating a hospitable space; (iii) exploring the questions; (iv) encouraging everyone’s contribution; (v) connecting diverse perspectives; (vi) listening together for patterns; and (vii) sharing collective discoveries [33, 40]. Prior to the start of the forum café table facilitators, who were a mix of health-care professionals and falls prevention researchers, were briefed and provided with information about the World Café’s principles and approach. Participants were welcomed to the forum, asked to sign the informed consent and complete the pre-forum questionnaires by staff and a group of student volunteers. Subsequently, the participants were invited to sit in groups of six to eight people at a table of their choice. Some participants were couples or friends, but most did not know each other. The research team’s main facilitator (A-MH), who was experienced in community participatory research, opened the forum with a welcome address and an introduction to the aims of the forum. A trained moderator facilitated the remainder of the forum.

The three prototype AV messages (which can be found here: 10.25917/5b3c2e51c22b3) intended to explicitly convey the following messages: i) falls can and will happen to anyone, and ii) prepare yourself for preventing a fall by doing activities that you enjoy. A short description of each AV message can be found in Appendix. The messages were played twice on two large projector screens, each followed by a 20-min conversation round. To stimulate conversation between participants semi-structured, open-ended questions and prompts were used. The questions were informed by the experiences gained during focus groups [30]. Table facilitators collected the participants’ responses on large paper sheets. Participants were also encouraged to jot their views onto post-it notes that were added to the collective responses. A random selection of the participants’ positive and negative responses was presented ‘live’ on the projector screens so participants could comment on and discuss what was said at other tables in the forum. During the conversation rounds video recordings were made by a roving staff-member to capture key discussions and the forum atmosphere. After each round the large paper sheets and post-it notes containing participant responses from each table were collected and collated by two experienced qualitative researchers (LDdJ, JF-C) while the table facilitator moved clockwise so that the next group could be exposed to a different set of semi-structured prompts and questions to the next table of participants. After the final conversation round the participants were asked to complete the post-forum questionnaires. Additionally, participants were asked which of the three AV messages they preferred, and if they had any further comments. After a break in which refreshments were served, the main facilitator summarized the key responses of each conversation round which were presented to the group on large summary paper sheets. This gave the forum participants the opportunity to provide any further input and feedback and also served as a form of member checking of the collective perspective before the forum concluded.

### Data analysis

#### Quantitative data

Likert-scale items from the questionnaire were summarised descriptively using frequencies (percentages). Based on the the total number of predetermined correct answers to the six selected multiple-choice statements combined (range 0–6) a composite ‘knowledge score’ was calculated for each participant. The differences between the participants’ composite knowledge scores and the median VAS scores (range 0–100) before and after the forum were analysed using a Wilcoxon signed rank-test. The significance level for all analyses was set at 0.05. All quantitative analyses were performed in IBM SPSS Statistics for Windows (Version 23.0; IBM Corp, Armonk, NY).

#### Qualitative data

Participants’ open responses from summary sheets reported by the table hosts, comments written on post-it notes, questionnaire and completed forum evaluation forms were transcribed and imported into Microsoft Excel. Multiple one-minute video-files and one 2-h audio-file recorded during the event were used to produce field notes. Written data was thematically analysed using a deductive approach [41] by three independent researchers (LDdJ, JF-C, A-MH) who were present throughout the forum. Firstly, two researchers (LDdJ, JF-C) familiarized themselves with all written data. The deductive approach was adopted for the data resulting from the conversations about the three AV messages. As responses about the AV messages had been identified in a prior study [30] a similar preliminary coding scheme was constructed. Four of six existing model components of this scheme (i.e. psychological capability-education; automatic motivation-persuasion; reflective motivation-persuasion; opportunity-persuasion) were derived from the COM-B (Capability, Opportunity and Motivation to undertake a health behaviour) framework [42]. Data was organized in codes under one of these three main themes in a stepwise categorization process. A third researcher (AM-H) independently coded and reviewed a sample of the data. Differences in coding and identification of candidate and final themes was discussed and agreed upon [41]. Representative notes from the table hosts and written comments from the participants were selected to exemplify the formation of subthemes [43]. The overarching theme was developed based on the summaries of the key responses of each conversation round and the member checking at the end of the forum. Themes developed during the post-forum thematic analysis completed this group consensus.

## Results

Thirty-eight older people participated in the forum. Their characteristics are presented in Table 1.

**Table 1:** Participant characteristics

Characteristic	*n* = 38
Age, years, mean (range)	71.4 (52–84)
Gender, female	27 (71)
Language spoken at home, English	37 (97)
Highest level of education	
Primary education	1 (3)
Secondary education or equivalent	16 (42)
Trade / technical / vocational training	9 (24)
University level education	12 (32)
Perceived general health	
Poor	2 (5)
Fair	5 (13)
Good	13 (34)
Very good	14 (37)
Excellent	4 (11)
Different types of prescription medication, mean (standard deviation)	2.7 (2.7)
Discussed falls prevention with GP or another health professional, yes	9 (24)
Ever undertaken any activities that are aimed at healthy aging or preventing falls?, yes	21 (55)
Experienced a fall in the past 12 months?, yes	15 (40)
Injured as a result of the fall?, yes	8 (53)

All data are frequencies (percentages) unless stated otherwise

### Quantitative results

Participants’ pre-forum responses to questionnaire items relating to the extent of their agreement or disagreement with statements about their capability and motivation towards undertaking falls prevention activities are presented in Table 2.

**Table 2:** Survey items relating to the participants’ pre-forum extent of agreement or disagreement with statements about their capability and motivation towards undertaking falls prevention activities

Survey item	Strongly agree	Agree	Undecided	Disagree	Strongly disagree
1. I think that I will fall over at some point in the next 12 months.	4 (11)	8 (21)	14 (37)	7 (18)	5 (13)
2. I worry that I could fall over.	7 (18)	18 (47)	2 (5)	9 (24)	2 (5)
3. If I were to fall over in the next 12 months I would be likely to get injured.	9 (24)	18 (47)	5 (13)	4 (11)	2 (5)
4. I am positive that if I try out physical activities that can prevent falls, it will reduce my risk of falling over.	22 (58)	13 (34)	5(5)	0 (0)	1 (30)
5. I intend to find out how I can reduce my risk of falling over.	23 (61)	14 (37)	1 (3)	0 (0)	0 (0)
6. If I fall over I will consider doing something to reduce my risk of falling again.	20 (53)	18 (47)	0 (0)	0 (0)	0 (0)
7. I will consider doing something to reduce my risk of falling over if other people whose opinions matter to me (such as family, friends, GP) think it is a good idea for me to do.^a^	17 (46)	16 (43)	2 (5)	1 (3)	1 (3)
8. I intend to do something to reduce my risk of falling over if I am offered the opportunity.^a^	19 (51)	14 (38)	2 (5)	1 (3)	1 (3)
9. I have never fallen over. Therefore I don’t need to do something to reduce my risk of falling over.^b^	0 (0)	2 (6)	1 (3)	18 (51)	14 (40)

^a^*n* = 37, ^b^*n* = 35

Table 3 shows that there were significant differences between the participants’ pre-forum and post-forum composite ‘knowledge’ scores and VAS scores.

**Table 3:** Pre- and post forum results of the 38 participants

Characteristic	Pre-forum	Post-forum	Wilcoxon signed rank-test	Difference
Knowledge scores^a^, *median (IQR)*	3 (2 to 4)^b^	5 (3 to 6)^c^	Z = −4.14	*p* = < .001
VAS scores, *median (IQR)*	90 (60 to 100)^c^	90 (70 to 100)	Z = − 2.41	*p* = .016

^a^The composite ‘knowledge score’ was based on the the total number of predetermined correct answers to six selected multiple-choice statements combined (range 0–6), ^b^*n* = 33, ^c^*n* = 37

The majority of participants responded positively to most of the questionnaire items relating to the three AV messages (Table 4), but there were also many who responded negatively or who were undecided.

**Table 4:** Survey items and response frequencies (percentages) regarding the 38 regaring the participants’ post-forum extent of agreement or disagreement with statements about the three AV messages

Survey item	Strongly agree	Agree	Undecided	Disagree	Strongly disagree
1. The videos had clear image and sound.	6 (16)	21 (55)	3 (8)	7 (18)	1 (3)
2. The videos made sense to me.	7 (18)	16 (42)	3 (8)	12 (32)	0 (0)
3. Now that I have seen these videos I know more about falls.	9 (24)	10 (26)	3 (8)	14 (37)	2 (5)
4. I think people will like these videos.	4 (11)	10 (26)	16 (42)	8 (21)	0 (0)
5. I found the videos boring.^a^	2 (6)	5 (14)	8 (22)	16 (44)	5 (14)
6. I think the videos appeal to all ages.	3 (8)	10 (26)	8 (21)	15 (40)	2 (5)
7. I think the videos are mainly for people who are over 75.	3 (8)	1 (3)	3 (8)	23 (61)	8 (21)
8. I would advise my friends to watch the videos.^b^	6 (16)	15 (41)	4 (11)	12 (32)	0 (0)
9. I can relate to the content of these videos.	4 (11)	20 (53)	4 (11)	10 (26)	0 (0)
10. I think that the videos have a powerful message.^b^	5 (14)	12 (32)	9 (24)	10 (27)	1 (3)
11. The videos were very serious.	4 (11)	6 (16)	8 (21)	17 (45)	3 (8)
12. The videos moved me.^b^	4 (11)	10 (27)	4 (11)	17 (46)	2 (5)
13. The videos inspired me to find out how I can reduce my risk of falling over.	10 (26)	11 (29)	8 (21)	8 (21)	1 (3)
14. I found the videos fun to watch.^b^	3 (8)	19 (51)	5 (14)	10 (27)	0 (0)
15. The messages that the videos gave surprised me.	5 (13)	11 (29)	7 (18)	13 (34)	2 (5)
16. The message(s) in the videos stood out to me.	6 (16)	9 (24)	9 (24)	14 (37)	0 (0)

^a^*n* = 36, ^b^*n* = 37

Twenty-one participants (58%) liked AV message 3 best and 7 (19%) preferred AV message 1.

### Qualitative findings

**Fig. 1:**
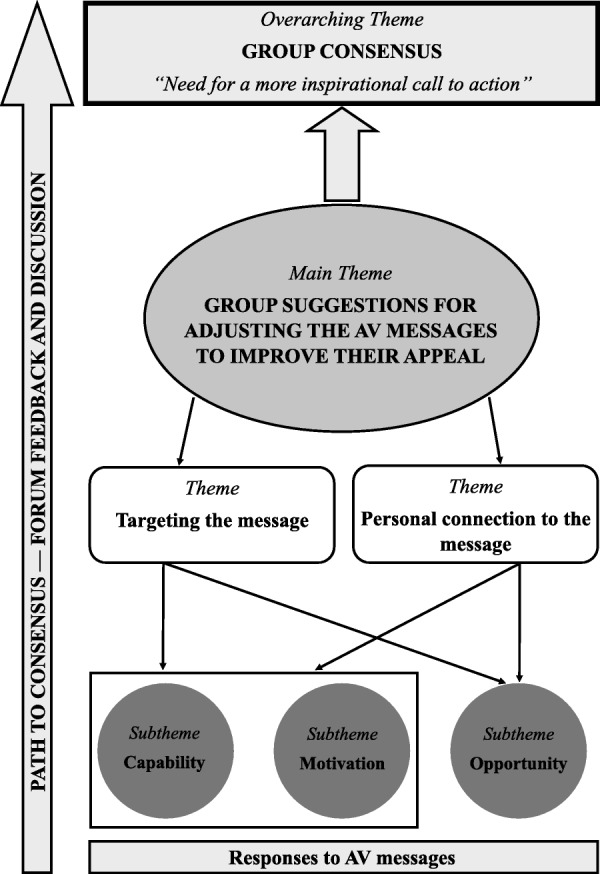
The path to consensus during the World Café forum

The final overarching perspective from the forum and the path to consensus is presented in Fig. 1. The final matrix demonstrated how diverse perspectives and responses from the group towards the AV messages were synthesized to provide a final group recommendation about the next steps to be taken.

### Group consensus: the need for a more inspirational call to action

The overarching theme from the forum was that the AV messages lacked persuasiveness. This theme was clarified and exemplified by the final collective perspective of the forum, where there was a strong consensus that the messages needed a more inspirational call to action. This was expressed by one participant who stated to the forum “*I enjoyed watching but didn’t feel more motivated.”* The forum responded with strong affirmation of this statement*.* Another participant summarized their response to the forum as, “*the video was too general to me so it didn’t motivate me*.”

### Improving the appeal of the messages

The main theme synthesized divergent ideas from each table and was that the messages should be adjusted in a manner that increased their appeal. This theme contributed to the overarching theme of making the messages more inspirational. For example one participant stated that the messages needed improvement because it made them “*think about falls seriously, but [it had] no guidelines*.” Two themes were identified about how the messages could be improved: i) targeting the message and ii) increasing personal connection.

### Targeting the message

Participants agreed that the messages needed to be tailored to reach a range of people with differing ages, abilities and lifestyles, because “*falls can happen at all ages.*” It was generally agreed that the message would be particularly ineffectual for younger older people as they “*feel bulletproof*” and don’t think falls prevention is relevant to them. One participant said: “*I don’t know if there’s anything that would motivate a 55+ year old who hasn’t fallen to seek further advice. [It] has to be a motivating personal factor.”* Two subthemes, capability and motivation, were identified. Some participants agreed the messages were well targeted because they increased their knowledge of how to prevent falls (psychological capability-education). For example, one participant stated: “*it made me aware falls can happen.”* Participants’ comments about exercise suggested that this message was portrayed effectively, with statements such as *“[it] puts message about more exercise across.”* However, there were divergent views with others saying the targeting could be improved by giving more and clearer information, thus improving cabability. For example, one suggested it would be “*better to have voice-over of contact info that appears on screen at the end.”* The main comments regarding the AV messages were that they did not provide enough ready-to-use falls prevention information*.* For example, participants responded that the messages were *“too general.”* Some responses suggested that people would *“respond better to more practical images”* and could use a *“more relevant exercise to prevent falls”.*

It was broadly agreed that the messages needed to raise motivation to have more impact on older people as the target audience. For example, it was generally agreed upon that the messages *“should have shown a fall”* and this would have given it more impact. One participant stated: “*We are a part of a generation of survivors. Be independent. It’ll be all right,”* suggesting that a targeted message should challenge the notion that falls only happen to “other people” and stress that people over the age of 60 have an increased falls risk.

### Personal connection to the message

Participants also indicated they wanted to feel a personal connection with the messages. They felt this was important because “*people think falls won’t happen to them”* and that feeling a personal connection with the message would make it resonate with the viewer. Several participants indicated they did not feel a personal connection to the messages because they had not had a fall and for that personal connection to be established “*It would need to happen to them first.”* Participants expressed that a personal connection with the message was also critical to raising motivation. Messages were viewed as easy to connect with if they aimed to reduce stigma associated with aging (*“being older isn’t a bad thing”)* and portrayed a positive view of aging (“*loved [the] smiles, lights, music, everyone having fun”*). For example, the messages in video 3 that *“portrayed fun [and the] social aspect of exercise”* were considered to be effective, as were messages that were considered *“clear, factual and not patronising.”* However, not all participants agreed that the messages were positively framed. Some thought that the messages were negative and patronizing. For example, one participant explained that video 1 was “*Too much like an ad. Boring. We are bouncy, alive”* and another explained that video 2 “*was too doom and gloom, not a positive aging message*”.

The third and final subtheme identified was opportunity. This was conceptualised within the COM-B framework as referring to older people having been prompted to seek out more information about falls prevention as suggested in the AV messages. The messages suggested visiting a website and / or speaking to their General Physician (GP). Several participants said they “*don’t have a computer*” and others expressed ambivalence about going online for more information, with one participant commenting that “*it can be a nuisance when a website keeps contacting you after you visit.”* Only a few participants said that they had been online to look at falls prevention information. Likewise, suggestions to visit the GP were also met with ambivalence. For example one participant stated that “e*ven though I didn’t like the ad, it did raise awareness and likelihood of speaking to GP about it, or seeking out info”* but another stated that they “*would never go to doctor before falling to talk about falls prevention*.” In order to increase opportunity to seek out falls prevention information participants suggested alternatives to online information such as advertising in newspapers and having printed information in places in the community.

## Discussion

This forum of older people gave a collective perspective on three prototype AV falls prevention messages. The collective perspective was that the messages needed to be more appealing and to inspire a clearer call to action in order to raise falls prevention motivation. The forum suggested this could be achieved by means of targeting the message and increasing the personal connection. The results also suggested that the forum participants had learned something about falls, thus increasing the participants’ falls prevention capability. In order to increase opportunity to seek out falls prevention information the forum suggested community-wide advertising.

The results of this World Café forum should be viewed together with the results of a previous study [30] during which participants of focus groups offered their feedback on the same prototype AV messages. The overarching theme of that study (“*we all look at things different ways”*) echoed the participants’ wide variety in positive and negative opinions about the messages. A similar variety in opinions was found during this study, but the current results additionally suggest that watching the AV messages had increased many of the participants’ falls prevention capability (knowledge and awareness) regardless of several participants not liking the messages for various reasons. This was a positive finding because, according to the COM-B health behavior change framework, having the capability is one of the three key elements fundamental to health behaviour change [23]. The results further suggested that watching the AV messages positively influenced some participants’ motivation to take up falls prevention activities. The finding that the group as a whole had significantly increased their VAS levels of intent to engage in healthy aging and falls prevention activities was probably due to the small sample. Despite these positive findings, participants had diverse opinions about the persuasiveness of the AV messages. Concerted efforts to improve the messages in cooperation with older people, as well as efforts to evaluate whether revised versions of the AV messages would be more motivating to older people to take action to do something about their falls risk, have yet to be undertaken.

If falls prevention messages are to positively influence falls prevention capability and motivation among other community-dwelling older people, they arguably have to contain remarkable educational facts and be strongly persuasive. Most efforts to date to educate the public about falls and persuade older people to do something about their falls risk have not been very successful. For example, research has shown older people have very limited falls prevention knowledge and motivation [16, 24, 44]. Even after having waged an 18-month multi-media campaign aimed at reducing falls, only 22% of older people that were surveyed from the campaigns’ most intensive coverage area had become more physically active [45]. There may be many reasons why older people cannot and do not want to engage in physical activities that are also good for falls prevention, for example because of health related issues and psychological reasons [46]. However, another reason may be that, to date, falls prevention calls to action have not been inspirational enough to people who are at a higher falls risk. This may be because community-dwelling older people, those who these messages specifically concern, have not been sufficiently involved in developing falls prevention messages calling for action. On the other hand, this research has shown that the AV messages brought out quite diverse perspectives despite the fact that they were developed by people from the target group. This finding suggests that it is important to involve a larger variety of older people to allow for a sufficient variety in message designs which are ‘tailored’ to a larger range of individuals. For example, participants suggested showing different kinds of physical exercise activities for different age groups in the AV messages such as dancing for the ‘younger’ older adults and gardening for the ‘older’ older adults. The questions remains, however, whether it is realistic to design such a broad range of messages or whether it might be more effective and efficient to design a layered, coordinated approach where there is both a role for mass media and individualisation of the messages by a health professional. Future research would have to be carried out to investigate what the best approach would be, and whether such a larger variety in messages’ designs with stronger calls to action would persuade older people to take concrete actions to do something about their falls risk such as take up healthy activities or falls prevention exercises.

The majority of participants of the current study reported they would not seek their falls prevention information using digital resources but rather prefer receiving falls prevention information via posters or brochures. Such a preference has been found previously [47, 48]. This suggests that the people over the age of 65 are still less likely to be online than younger people [49, 50] and that not using computers may act as a barrier for older people in finding falls prevention information. A question regarding alternatives to online falls prevention resources were in concordance with findings from a previous World Café during which participants reported they would rather seek their falls prevention information in community places such as the doctor’s surgery, libraries, community centres and shopping centres [24]. These findings confirm that older people predominantly rely on active participation in the local community to obtain information about issues related to health [51]. It is of note that the findings of the current studies are based on only one component (i.e. AV messages) that can be used during a falls prevention marketing trial or campaign. Previously it has been suggested that is important to disseminate the falls prevention information through multiple channels, if possible with distribution of free or reduced-price health-related products [52]. During a community-wide falls prevention campaign several inspirational components could be used at the same time. For example, a wide range of AV messages (on television) could be followed-up with remarkable advertisements in newspapers, on buses and on the radio coupled with offering incentives for people to attend (free) falls prevention or other aging related services. Future population-based research would need to be carried out to assess whether such a multi-media falls prevention campaign could help to increase in falls prevention knowledge and boost the uptake of falls prevention activities among community-dwelling older people.

### Strengths and limitations

A major strength of this study is that the findings from one large group’s perspective confirms the findings of a similar study during which smaller focus groups were used. By using a different method, this study has facilitated validation of data through cross verification, hence increasing the transferability of the findings for community-dwelling older people. The group of participants also seemed to mirror a community-dwelling older adult population with 40% of the attendees having had a fall in the past 12 months. On the other hand, this means the majority (60%) of the forum participants had not experienced a fall. Previous research has shown that it is difficult to convince older adults, who have not personally experienced a fall, that taking up falls prevention strategies is important [16, 24]. Therefore, this subgroup may have influenced the overall nature of the responses during the forum. Further limitations of this study include an underrepresentation (29%) of male participants and participants with different cultural backgrounds and health literacy levels, who may also have offered a different perspective. Although the location of the venue was considered central, participants were required to travel either by car or public transport. This may explain why only younger, fitter participants attended the forum. The participants may have been more proactive to provide their thoughts about falls prevention messages than the broad population of older adults living in the community, and may not have been representative of socio-economically disadvantaged individuals. It is also of note that the participants’ initial levels of motivation were surprisingly high (90 on a scale of 100) whilst the qualitative results suggested that motivation only increased for some. This suggests that some participants gave socially desirable responses.

## Conclusions

Feedback of older people on a series of three prototype AV falls prevention messages suggested that these had increased the participants’ falls prevention capability and positively influenced their motivation to take falls prevention actions. Despite these positive findings, group consensus was that a more inspirational call to action was needed. A wider variety of revised and tailored AV messages, as one component of a community-wide falls prevention campaign, could be considered in an effort to persuade older people to take decisive action to do something about their falls risk. Promoting falls prevention information should be focused in community places rather than using online resources.

## Data Availability

The video files used during the current study are available in the Research Data Australia repository, 10.25917/5b3c2e51c22b3. The raw data used and/or analysed during the current study are available from the corresponding author on reasonable request.

## Appendix

### Short descriptions of the three prototype audio-visual (AV) messages

#### Prototype AV Message 1 (‘Family Life’)

AV message 1 (duration: one minute and 28 s) shows a baby laughing and then crying, a young girl learning to ride a bike with her mother and then sitting at a table with a plaster cast around her arm, a young adolescent being tackled playing Australian football and a young adult in pain rubbing her twisted ankle and stepping onto a step with her foot in a plaster cast, respectively. During this sequence a gentle voice-over states that sometimes we fall and hurt ourselves but that falling is a part of growing up; that life is full of tumbles and that nobody goes through life without taking their fair share of knocks and falls, but that falls happen more as we get older. In the next scene all the same characters, including their grandparents, are enjoying a family picknick in the park while grandfather is playfully throwing a football with his granddaughter. The voice-over states that, even if one falls down, it should not stand in the way of continuing one’s life journey. The voice-over then continues saying that one in three people over 60 fall, that falls happen more as we age, but that they can be prevented. The voice-over encourages people to do something about their falls risk before they are 60, and to not let a fall stand in the way of living a happy life. The message concludes with the frames *“This message was developed for older people. By older people”* and with the written text that encourages the viewer to find out more about falls prevention by going to a (fictitious) website / phone number.

#### Prototype AV Message 2 (‘Kitchen’)

AV message 2 (duration: 45 s) shows an older man standing in the kitchen by the sink washing dishes. As he turns around to the stove he loses his balance. He stumbles, but he manages to support himself just enough on the kitchen bench to avoid landing on the floor before regaining his balance. During this sequence a voice-over asks the viewer what causes more than 98.000 people over the age of 65 to be admitted to hospital each year, and in older people causes 3000 deaths in one year alone which is more than 10 times the number of older people who die from a car accident. In the next frames the voice-over reads out the on-screen statements that up to 60% of people over the age of 65 that fall over injure themselves, and that most falls happen inside the home. In the next scene the older man’s granddaughter walks into the kitchen, and the main character jokingly throws her a towel. During this sequence a voice-over states that the good news is that falls are preventable. The message concludes with the voice-over reading out the on-screen text:” *Falls prevention. Find out more. This message was developed for older people, by older people.”* At the same time a (fictitious) website and phone number is shown.

#### Prototype AV Message 3 (‘Dance’)

AV message 3 (duration: one minute and 32 s) starts with the main character Brian sitting in the GP’s waiting room. The GP calls him in. At the end of his appointment Brian is asked if there is anything else she can do for him. Brian worryingly replies that he tripped and nearly fell in the kitchen yesterday. The GP thinks for a moment, responds by saying she thinks she knows exactly what he needs, and writes out a prescription. The next frame is an on-screen statement that says that each year 1 in 3 people over the age of 60 fall over. The subsequent scene shows Brian looking at his prescription in confusion and handing it over to an impressively muscular man in a doorway, whilst muffled music is pumping and lights are flashing in the background. Once inside the music level swells….. The next sequence shows a variety of older people dancing whilst an enthousiastic elderly lady DJ is manning the turntable. Then a sequence of on-screen statements state *“Whether you choose dancing or otherwise…one of the best ways to reduce the risk of falling over…is to exercise”*. Brian first looks confused, but once the DJ makes eye contact with him and points to him, Brian realises what it is all about and ends up with a big smile on his face. An on-screen statement follows, stating” *You should start thinking about fall prevention methods when you reach 55.”* The message concludes with the text *“This message was developed for older people. By older people”* and with the written text that encourages the viewer to find out more about falls prevention by going to a (fictitious) website / phone number.

## Abbreviations

AV: Audio-visual
COM-B: Capability, Opportunity and Motivation to undertake a health behaviour
GP: General physician
ICC: Intraclass correlation coefficient
VAS: Visual analogue scale
